# Supplementary material for: Prevalence of dental caries and associated risk factors among People Living with HIV/AIDS and HIV uninfected adults at an HIV clinic in Kigali, Rwanda
Source: PLoS One. 2023 Apr 6;18(4):e0276245. doi: 10.1371/journal.pone.0276245 (PMC10079010; doi:10.1371/journal.pone.0276245)
Supplement: S1 Table — (PDF) [file pone.0276245.s001.pdf]

Table 1: Description of participants' behaviors factors

Variables	Frequency	Percentage
**Brushing**		
Yes	398	99.5
No	2	0.5
**Frequency of brushing**		
Once or more a day	363	91.2
Once or more a week	21	5.3
Once or more a month	14	3.5
**Use of toothpaste**		
Yes	389	97.2
No	11	2.8
**Last visit to the dentist**		
Never received dental care	61	15.25
Less than 6 months to 1 year	72	18.00
More than 1 year but less than 5 years	104	26.00
5 years and more	163	40.75
**Reasons for last dental visit**		
Never visited dentists	163	40.7
Consultation/advice	18	4.5
Pain or trouble with teeth, gums or mouth	194	48.5
Routine check-up/treatment	25	6.3
**Reasons not visiting dentists**		
Visited dentists	238	59.5
Reasons related to fear, negligence, self-treatment and traditional healers help	30	7.5
Reason related to affordability and lack of time	31	7.8
Never experienced dental problems	101	25.2
**Experienced oral diseases related discrimination**		
Yes	31	7.8
No	369	92.2
**Category of discriminating people**		
Not experienced oral diseases related discrimination	370	92.50
Discrimination from self ,relatives, friends and neighbors	30	7.50
**Disclosing HIV status to dentists**		
Yes	132	33.00
No	19	4.75
**Reason of none disclosing**		
Not applicable	385	96.25
Confidentiality and discrimination	15	3.75
**Frequency eating fruit**		
Several times a day or every day	66	16.50
Several times a week or once a wee	212	53.00

Several times a month or seldom/never	122	30.50
**Frequency eating biscuit**		
Several times a day or every day	18	4.50
Several times a week or once a week	84	21.00
Several times a month or seldom/never	298	74.50
**Frequency eating jum or honey**		
Several times a day or every day	21	5.25
Several times a week or once a week	44	11.00
Several times a month or seldom/never	335	83.75
**Frequency eating sweet gum**		
Several times a day or every day	10	2.50
Several times a week or once a week	33	8.25
Several times a month or seldom/never	357	89.25
**Frequency eating sweet candy**		
Several times a day or every day	8	2.00
Several times a week or once a week	33	8.25
Several times a month or seldom/never	359	89.75
**Frequency of taking soft drinks including lemonade, Coca Cola or other artificial juices**		
Several times a day or every day	8	2.00
Several times a week or once a week	33	8.25
Several times a month or seldom/never	359	89.75
**Frequency of taking tea with sugar**		
Several times a day or every day	222	55.50
Several times a week or once a week	85	21.25
Several times a month or seldom/never	93	23.25
**Frequency of taking coffee with sugar**		
Several times a day or every day	222	55.50
Several times a week or once a week	85	21.25
Several times a month or seldom/never	93	23.25
**Smoking**		
Yes	5	1.25
No	395	98.75
**Frequency of smoking**		
Not applicable	395	98.75
Several times a day	5	1.25
**Alcohol consumption**		
Yes	115	28.75
No	285	71.25
