# Supplementary material for: Prevalence of dental caries and associated risk factors among People Living with HIV/AIDS and HIV uninfected adults at an HIV clinic in Kigali, Rwanda
Source: PLoS One. 2023 Apr 6;18(4):e0276245. doi: 10.1371/journal.pone.0276245 (PMC10079010; doi:10.1371/journal.pone.0276245)
Supplement: S2 Table — (PDF) [file pone.0276245.s002.pdf]

Table 2. Description of other underlying conditions reported by participants

Variables	HIV+, N=200			HIV- N=200		
	**Yes** n(%)	**No** n(%)	**Not applicable*** n(%)	**Yes** n(%)	**No** n(%)	**Not applicable*** n(%)
Diabetes	10 (5.00)	190 (95.00)	-	7(3.50)	193(96.50)	-
Hypertension	20 (10.00)	180 (90.00)	-	13(6.50)	187(93.50)	-
Being pregnant	0 (0.00)	114 (57.00)	86 (43.00)	1 (0.50)	109 (54.50)	90 (45.00)

* Not applicable indicate the number of males because they cannot be pregnant
