# Supplementary material for: Prevalence of dental caries and associated risk factors among People Living with HIV/AIDS and HIV uninfected adults at an HIV clinic in Kigali, Rwanda
Source: PLoS One. 2023 Apr 6;18(4):e0276245. doi: 10.1371/journal.pone.0276245 (PMC10079010; doi:10.1371/journal.pone.0276245)
Supplement: Attachment — Submitted filename: Dental Caries in Rwanda - Comments.docx [file pone.0276245.s003.docx]

Prevalence of dental caries and associated risk factors among HIV-positive and HIVnegative adults at an HIV clinic in Kigali, Rwanda --Manuscript Draft-- Manuscript Number: PONE-D-22-27201Abstract b

Abstract

1. Line 17-19; “HIV-positive (HIV+) persons.” – Avoid hyphen between HIV positive and HIV negative phrase’s. Can be confusing as HIV-positive looks like a negative.
2. Line 22 – attending an HIV clinic
3. 3. Line 27 – Dental Caries were assessed using the WHO Decayed (D), ……
4. Line 31 – delete experience
5. Edit to “being female”

Introduction

1. Line 51-52 – five an example of these “systemic conditions”
2. Line 52 – citation does not describe Caries prevalence in HIV positive – it’s about stigma
3. Line 54 unclear

Materials and Methods

1. Line 88 - HIV voluntary testing and who was “were, Not was”
2. Line 100 onwards. I assume even HIV negative were outpatients. Please clarify
3. Among the HIV positive participants, was informed consent obtained, and if so when was this done? Also clarify in lines 131 and 132 regarding process of obtaining informed consent
4. Line 104 – revise term “old cases”
5. How were participant selected? Was it consecutive, or some other criteria was used
6. Was brushing teeth also considered as a factor.
7. What of soft drinks and sweets among the foods other than just sugar and fruits.
8. Table 4 – clarify – WHO staging for HIV

Results

1. Line 147 – what is “ubudehe” ??? – Only defined in line 191 for first time - ubudehe (income status),…
2. In table one – how would or did these differences in age, sex, education status etc. impact on prevalence of dental caries.
3. For example, are they generally lower in younger more educated persons? What does the literature say regarding these factors in table one in a general HIV neg for example population?

Were HIV positive living in urban middle class or urban low income earner settings (such as more of slum areas?

4. Line 190-192 unclear context. Please clarify on this statement regarding {frequency of eating fruits, frequency of drinking 192 tea with sugar and alcohol consumption} in the multiple regression analysis.

Discussion

1. The statistically significant differences between the HIV neg and HIV pos. populations seems to indicate that the two groups were not comparable. This makes if difficult to make meaningful conclusions of these results comparing the 2 patient populations. Could the controls be patients attending the dental clinic – who tested HIV negative rather than those attending the HIV clinic for an HIV test – who may be younger etc.
2. Data also showed that dental caries are more in older persons, and the HIV patients were significantly older.
3. Lines 215 to 216 – It’s important to consider that factors other than dental hygiene education in HIV clinics – to explain the higher dental caries among the HIV infected persons.
4. Could caries be more in women because of their health seeking behaviors?
5. Line 264 – women’s reproductive history was not determined, so difficult to attribute the higher dental caries to hormonal and reproductive factors.
6. Lines 276-277 –” The results of our study revealed that a higher prevalence of HIV + and HIV- adults who frequently visited dentists experienced more caries compared to participants who less frequently visited dentists.” It possible that those with cries may be more prone to dental caries – e.g. genetic or eating habits – so visit dentists more, rather than risk being higher – by visiting dentists.
7. Also poor health seeking behavior is not compatible with the data of more dental visits. Is it possible that the dental care given is sub optimal – this possibly more visits for the same (rather than recurring) dental caries?
8. The viral load issue seems to be the single most significant outcome of this study – yet its discussed last

Limitations and conclusions

1. It important to mention the limitation of the non-comparable HIV pos. and neg demographic characteristics.
2. Also the above differences may impact on the study conclusions

References

1. Ref 2 – use proper citation
2. Infact all reference citation needs to be improved to one format.
