# Supplementary material for: Prevalence of dental caries and associated risk factors among People Living with HIV/AIDS and HIV uninfected adults at an HIV clinic in Kigali, Rwanda
Source: PLoS One. 2023 Apr 6;18(4):e0276245. doi: 10.1371/journal.pone.0276245 (PMC10079010; doi:10.1371/journal.pone.0276245)
Supplement: Attachment — Submitted filename: Review.docx [file pone.0276245.s004.docx]

The association between HIV and dental caries is an interesting subject. Regarding the article, I have the following questions that I would like to discuss with the author.

1. Please state in the manuscript whether the HIV negative participants had any other underlying diseases or caries related diseases. Otherwise, the sample is not representative when illustrating the association between HIV and dental caries
2. As mentioned in line 110 and 111, “those who agreed to participate were sent to the researchers for consent signature and data collection”, how to obtain the data of the participants other than dental examination, please describe in detail? Is it a self-answering questionnaire, a one-on-one interview, or something else?
3. Please state in the manuscript which software and version you used for your sample size calculations. According to the prevalence of caries in HIV+ patients and HIV- participants, the ratio and the study power provided by the manuscript, the sample size I calculated by PASS15.0 was a little different from yours.
4. Are there any missing values or no responses in your 400 samples, and if so, how do you consider them when calculating the sample size?
5. As mentioned in line 120 and 121, “Multiple logistic regression tests were done to determine the relationship of dental caries (dichotomous outcome) with various factors”, I don’t really understand why **Multiple Logistics Regression** was used instead of **Binary Logistics Regression**, as you yourself said that dental caries was dichotomous outcome. Could you please identify the dependent variables in the regression analysis?
6. What software and version were used for the regression analysis? They need to be stated in the manuscript.
7. As mentioned in line 124 and 125, “Kappa score statistical analysis was used to evaluate the intra-examiner reliability during examiner calibration”, so what was the value of the Kappa score? Please state it in the manuscript.
8. Why the category 3 and the category 4 were described together in Table 1?

From your description of categories 3 and 4, I think their SES are very different and should not be combined. Actually, the prevalence or DMFT of dental caries is strongly correlated with SES. If the objective of the study is to discuss the relationship between HIV and dental caries, the more balanced the distribution of samples in SES, the more bias can be reduced.

9. Please describe what analysis was performed in Table 3. What indicator of dental caries was used in comparison with factors associated with dental caries? Is it prevalence? It needs to be described in the manuscript.
10. The meaning of the sentence in this line of 178 and 179 “Participants who visited dentists **after 1 year but less than 5 years**” is unclear.
11. I don't quite understand the analysis method used by the authors in Table 3. If it was a single factor Chi-square analysis, why were HIV-related factors such as viral load didn’t need to be included in this analysis, but only in the regression analysis? If it was the result from a logistic regression, the total associated factors also should be included and the results should be presented in one table including both the Crude Odds Ratio and the Adjusted Odds Ratio.
12. The fitting test result of the logistic regression model should be attached at the end of Table 4, so that readers can judge the reliability of the model.
